# Similarities and differences in the nucleic acid chaperone activity of HIV-2 and HIV-1 nucleocapsid proteins *in vitro*

**DOI:** 10.1186/1742-4690-11-54

**Published:** 2014-07-03

**Authors:** Katarzyna Pachulska-Wieczorek, Agnieszka K Stefaniak, Katarzyna J Purzycka

**Affiliations:** 1 Institute of Bioorganic Chemistry, Polish Academy of Sciences, Noskowskiego 12/14, 61-704 Poznań, Poland

**Keywords:** NCp8, NCp7, Nucleocapsid protein, Nucleic acid chaperone activity, HIV-1, HIV-2

## Abstract

**Background:**

The nucleocapsid domain of Gag and mature nucleocapsid protein (NC) act as nucleic acid chaperones and facilitate folding of nucleic acids at critical steps of retroviral replication cycle. The basic N-terminus of HIV-1 NC protein was shown most important for the chaperone activity. The HIV-2 NC (NCp8) and HIV-1 NC (NCp7) proteins possess two highly conserved zinc fingers, flanked by basic residues. However, the NCp8 N-terminal domain is significantly shorter and contains less positively charged residues. This study characterizes previously unknown, nucleic acid chaperone activity of the HIV-2 NC protein.

**Results:**

We have comparatively investigated the *in vitro* nucleic acid chaperone properties of the HIV-2 and HIV-1 NC proteins. Using substrates derived from the HIV-1 and HIV-2 genomes, we determined the ability of both proteins to chaperone nucleic acid aggregation, annealing and strand exchange in duplex structures. Both NC proteins displayed comparable, high annealing activity of HIV-1 TAR DNA and its complementary nucleic acid. Interesting differences between the two NC proteins were discovered when longer HIV substrates, particularly those derived from the HIV-2 genome, were used in chaperone assays. In contrast to NCp7, NCp8 weakly facilitates annealing of HIV-2 TAR RNA to its complementary TAR (−) DNA. NCp8 is also unable to efficiently stimulate tRNA^Lys3^ annealing to its respective HIV-2 PBS motif. Using truncated NCp8 peptide, we demonstrated that despite the fact that the N-terminus of NCp8 differs from that of NCp7, this domain is essential for NCp8 activity.

**Conclusion:**

Our data demonstrate that the HIV-2 NC protein displays reduced nucleic acid chaperone activity compared to that of HIV-1 NC. We found that NCp8 activity is limited by substrate length and stability to a greater degree than that of NCp7. This is especially interesting in light of the fact that the HIV-2 5′UTR is more structured than that of HIV-1. The reduced chaperone activity observed with NCp8 may influence the efficiency of reverse transcription and other key steps of the HIV-2 replication cycle.

## Background

HIV infection in humans can be caused by two viruses: HIV-1 and the less pathogenic HIV-2 [1,2]. HIV-2 is more closely related to SIV_MAC_ and has only limited genome and protein sequence identity with HIV-1. However, HIV-1 and HIV-2 share a similar genome organization, virion structure and replication cycle. Like other retroviruses they possess a dimeric genome assembled from two identical RNA sense strands interacting near their 5′-ends. In the mature viral particles and during early steps of the replication cycle the genomic RNA is extensively coated by ~ 2400 copies of the nucleocapsid protein, derived upon proteolysis of Gag precursor polyproteins [3,4]. This structural role is only one of a multitude of functions performed by these proteins. The nucleocapsid domain of Gag and mature nucleocapsid protein (NC) are involved in critical steps of HIV replication, such as primer tRNA annealing, reverse transcription, vRNA dimerization and packaging, virion assembly and proviral integration into host DNA [5-7]. Many of those functions are correlated with the ability of NC to act as a nucleic acid chaperone (NAC). Such chaperone proteins bind nucleic acids with broad specificity and facilitate their folding by destabilizing misfolded, kinetically trapped structures and enabling the formation of the thermodynamically most favored form [6-9]. They do not require ATP and their binding is no longer required once the most stable nucleic acid structure is reached [9,10].

**Figure 1:**
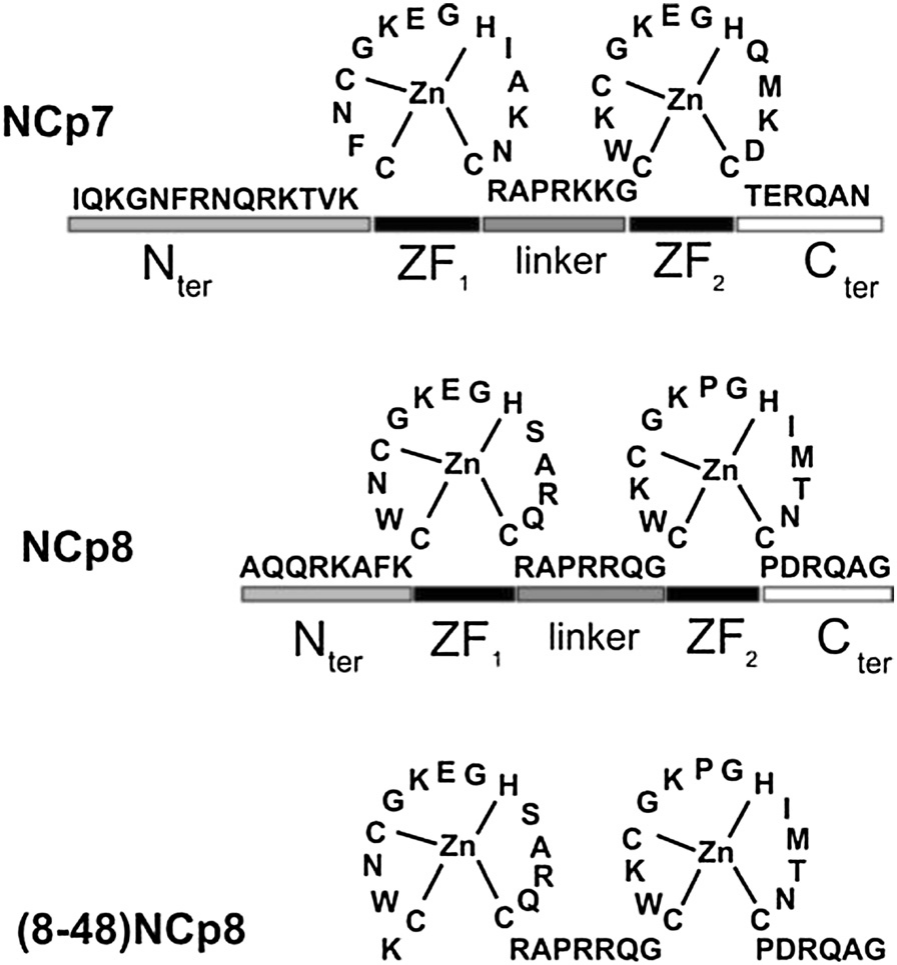
**The nucleocapsid protein of HIV-1**_**NL4–3 **_**(NCp7), HIV-2**_**ROD **_**(NCp8) and the truncated NCp8 mutant ((8–48) NCp8).**

The fully processed, mature nucleocapsid proteins of HIV-1 (NCp7) and HIV-2 (NCp8) are small basic proteins sharing 67% similarity in amino acid sequence (Figure 1). They contain two strictly conserved Cys-X_2_-Cys-X_4_-His-X_4_-Cys (CCHC) zinc finger domains (ZFs) that are linked by a short basic amino acid sequence (linker region). In both proteins the ZFs are flanked by a short C-terminus and basic N-terminal domain. The N-terminal region of NCp8 is markedly shorter than that of NCp7 and consequently NCp8 is a 48-amino acid protein, whereas NCp7 contains 55 aa [11-13]. Except for the structured ZFs, retroviral NC proteins are highly flexible in their free form. However ordering of NCp7 protein structure has been shown upon binding to nucleic acid, where the disordered N-terminus of the protein forms a 3_10_ helix [14,15]. The N-terminal domain of NCp8 is too short to form similar helix and limited structural information suggests that the NA recognition mechanism for NCp8 is different and the flexible second ZF plays role similar to that of NCp7 N-terminal region [16,17].

**Figure 2:**
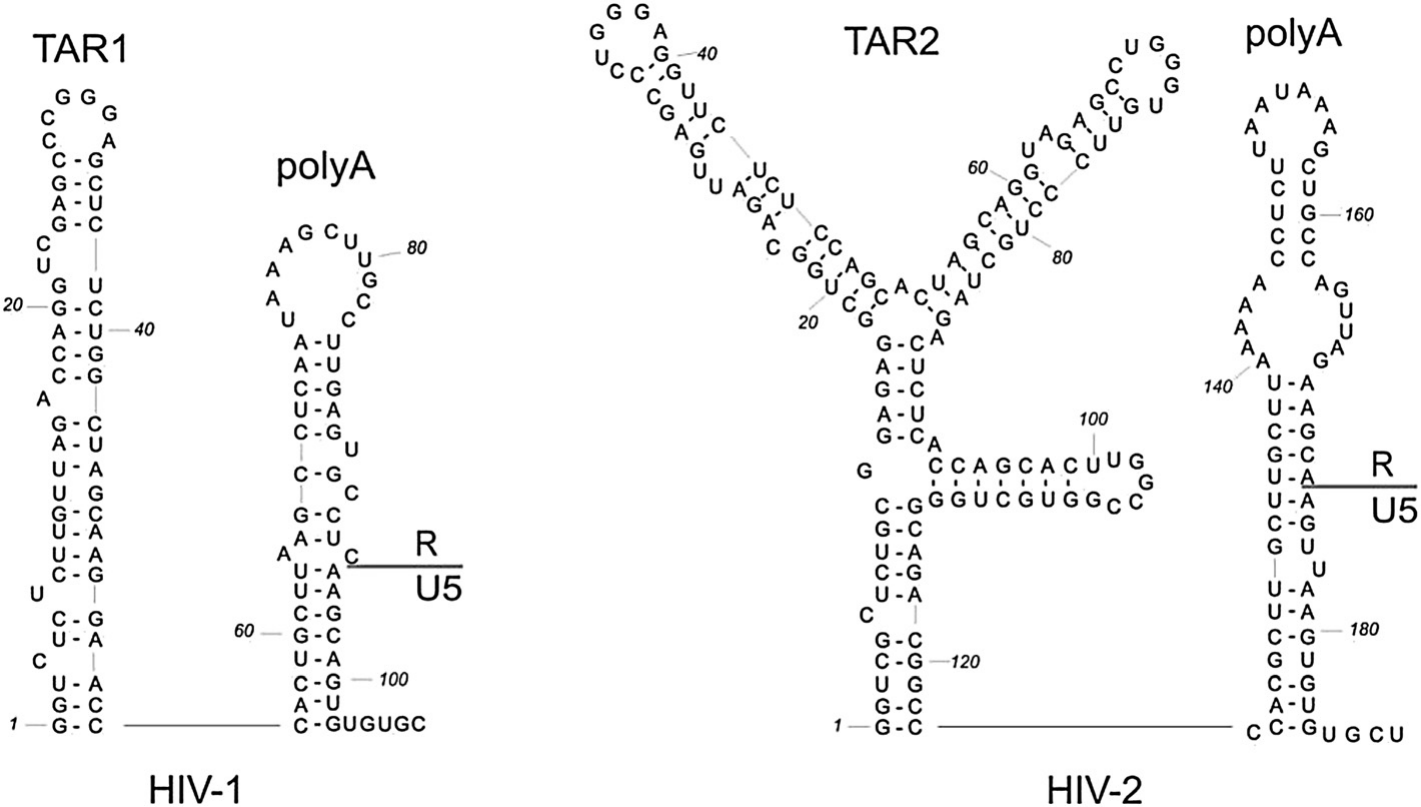
**Secondary structure models of the HIV-1**_**MAL **_**and HIV-2**_**ROD **_**RNA 5′ R regions.**

**Figure 3:**
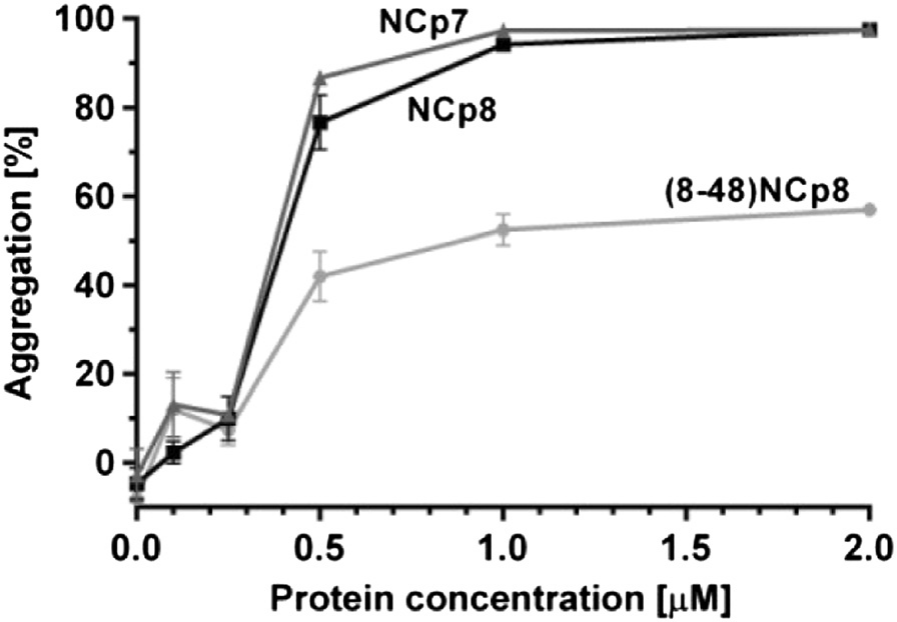
**Comparison of NCp7, NCp8 or (8–48) NCp8 aggregation activity.** Percent TAR1 (−) aggregated by each protein. Assays were performed as a function of protein concentration as indicated. The graphs represent averaged data from three independent experiments. The error bars represent standard deviations.

Due to the pleiotropic effect of mutations, testing the protein NAC activity in cell culture is difficult. Therefore most studies are based on different experimental *in vitro* models, which mimic the steps of retroviral replication, such as human tRNA^Lys3^ annealing to viral RNA or diverse strand transfers of reverse transcription. The cellular tRNA^Lys3^ is packaged into nascent virions and serves as a primer for reverse transcription of HIV RNA [18]. NCp7 chaperones the annealing of an 18 nt fragment at the 3′ end of tRNA^Lys3^ to a complementary primer binding site (PBS) sequence located within the highly structured 5′-UTR region of the HIV-1 genome [5,19]. The reverse transcriptase extends the primer tRNA^Lys3^, copying the U5 and R regions and synthesizing the complementary (−)ssDNA strand. Newly synthesized (−)ssDNA must be transferred from the 5′ R region (Figure 2) to 3′ R region of the viral genome to resume elongation; this process is known as first strand transfer [20]. In the case of HIV-1, NCp7 chaperones this process and promotes annealing of (−)ssDNA to the complementary 3′ R sequence [5,21].

NAC activity of HIV-1 nucleocapsid protein (NCp7) has been extensively studied and numerous efforts have been undertaken to understand the molecular mechanism of its activity. *In vitro* studies have shown nucleic acid aggregation, destabilization, and rapid binding kinetics are key characteristics of NA chaperones, including NCp7 [9,13,22,23]. It is known that basic amino acids residues (Arg and Lys) of the N-terminal and linker domain are involved in sequence nonspecific aggregation and annealing of nucleic acids, whereas NCp7 zinc fingers are essential for sequence specific binding and duplex NA destabilization [24-26]. Recent *in vitro* studies have demonstrated that NC proteins from several retroviruses (HIV-1, MuLV, RSV and HTLV-1) display non-equivalent levels of nucleic acid chaperone activity [27-29]. In light of this information, it is particularly interesting to compare the NAC activity of nucleocapsid proteins from HIV-1 and HIV-2.

This study is the first to characterize the *in vitro* nucleic acid chaperone activity of the HIV-2 NC protein and to compare it with HIV-1 NC. Biochemical assays with substrates derived from the HIV-1 and HIV-2 genomes were performed to compare the ability of both proteins to chaperone nucleic acid aggregation, annealing and strand exchange in duplex structures. Using a truncated NCp8 mutant, we found that the short, basic N-terminal domain is crucial for NCp8 activity. NAC activity of NCp8 and NCp7 in assays with HIV-1 TAR oligonucleotides was similar, but when longer HIV-1 substrates or particularly those derived from the HIV-2 genome were used, interesting differences between these two proteins were discovered. In contrast to NCp7, NCp8 weakly facilitates annealing of HIV-2 TAR RNA to complementary TAR (−)DNA. Moreover, NCp8 was unable to efficiently stimulate tRNA^Lys3^ annealing to its respective HIV-2 PBS motif. Our data suggest that the NAC activity of HIV-1 and HIV-2 NC proteins is not equivalent and NCp8 exhibits lower chaperone activity *in vitro* than NCp7.

## Results

The chaperone proteins may act in the sequence independent manner; therefore oligonucleotides derived from HIV-1 genome are usually used in chaperone assays. In this study we used DNA and RNA oligoncleotides derived from HIV-1 and HIV-2 genome (Figure 2), marked TAR1, R1 and TAR2, respectively.

### The N-terminal region contributes to the nucleic acid aggregation activity of NCp8

The ability to cause non-specific aggregation of nucleic acids is considered one of the principal components of the NAC activity of retroviral nucleocapsid proteins [11,12,30]. In the case of NCp7, basic residues in the N-terminal domain were shown most important for nonspecific interaction with and aggregation of nucleic acids and also annealing activity [12,31]. Interestingly, a major difference between NCp7 and NCp8 at the amino acid sequence level is the significantly shorter N-terminal region of NCp8 (Figure 1). Whereas the NCp7 N-terminal segment contains 14 aa residues including 2 Arg and 3 Lys, the corresponding region of NCp8 is composed of 8 aa and 1 Arg and 2 Lys are present.Sedimentation assays were used to directly examine NA aggregation properties of NCp8 and to compare them to that of NCp7. The TAR1(−) DNA/ and TAR1(+) DNA were incubated with increasing concentrations of NCp8 or NCp7. Aggregates were pelleted by centrifugation, whereas free NA and proteins remained in the supernatant. Both NC proteins effectively aggregated NA at 0.5 μM concentration (protein to nt molar ratio 1:11) (Figure 3). However, the maximal level of aggregation was observed upon saturated binding of NA at 1 μM protein concentration (1 NC per 5.6 nt). Accordingly, despite differences in the number of the basic residues and composition of their N-terminal domains, NCp7 and NCp8 display very similar nucleic acid aggregation properties.To directly exploit the role of the NCp8 N-terminal segment, we performed sedimentation assays with a truncated NCp8 peptide: (8–48) NCp8, lacking 7 aa residues from the protein N-terminus (Figure 1). Results obtained indicated that (8–48) NCp8 displayed reduced aggregation properties. Only ~ 50% of NA aggregation was detected at saturating peptide concentration.

### Comparison of the nucleic acid annealing activity of NCp8 and NCp7

Proteins considered to be NA chaperones are able to facilitate annealing of complementary or nearly complementary nucleic acids [10]. In the present study, two different experimental models were used to assay the *in vitro* annealing activity of HIV-1 and HIV-2 NC proteins. The first model mimics the annealing of the (−)ss DNA strand and acceptor RNA at the viral 3′ UTR during the first strand transfer of reverse transcription. The second corresponds to the placement of tRNA^Lys3^ on primer binding site (PBS) of HIV-2 RNA. The annealing activity of proteins was measured using gel mobility shift assays.

#### TAR and R annealing assays

**Figure 4:**
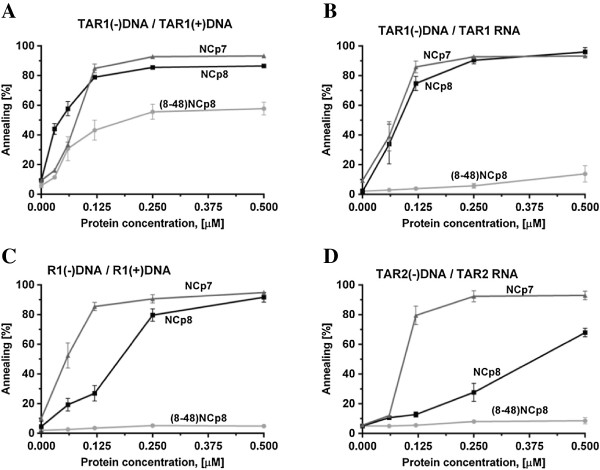
**Comparison of NCp7, NCp8 or (8–48) NCp8 annealing activity.** The assays were performed using different DNA and RNA substrates as a function of protein concentration as indicated. Annealing of TAR1(−)DNA/TAR1(+)DNA **(A)**, TAR1(−)DNA/TAR1 RNA **(B)**, R1(−)DNA/R1(+)DNA **(C)**, TAR2(−)DNA/TAR2 RNA **(D)**. The graphs represent averaged data from three independent gel shift annealing experiments for each protein. The error bars represent standard deviations.

A comparison of the NCp8 and NCp7 induced annealing of TAR1(−) and TAR1(+) DNA showed that both proteins effectively accelerated the formation of DNA duplex. (Figure 4A). More than 80% of TAR1 DNA oligonucleotides were present in duplex form at saturating NCp8 or NCp7 concentrations, corresponding to protein to nt molar ratio 1:6.3 (0.125 μM). This is in agreement with previous reports showing that saturated levels of protein, demonstrated to be 1 protein per 5–8 nt, is required for optimal NCp7 chaperone activity [22,27,30,32]. Moreover, NCp7 and NCp8 displayed comparable, high annealing activity of TAR1(−) DNA and TAR1 RNA (Figure 4B). Interestingly, we observed a difference between NCp7 and NCp8 in assays utilizing longer HIV-1 R region oligonucleotides (Figure 4C). The concentration of NCp7 required for effective annealing of R1(−) and R1(+) DNA was lower than that of NCp8. Almost 90% of R1 strands were annealed at 0.125 μM NCp7 but only ~ 30% at the same concentration of NCp8. However at saturating NCp8 or NCp7 concentrations (0.25 μM, 1NC per 5.3 nt) the R1(−)/R1(+) annealing was close to 90% for both proteins. The R1 oligonucleotides are longer than TAR1 (96 nt vs. 56 nt) and in addition to the TAR hairpin, a second short poly(A) hairpin may be forming at the 3′ end. To further compare NCp8 and NCp7 activities we performed annealing assays with TAR2 oligonucleotides (123 nt) derived from HIV-2 RNA (Figure 2). The TAR2 RNA forms a more complex structure than TAR-1 RNA [33,34]. We observed that NCp8 was not able to effectively anneal TAR2 RNA and TAR2(−) DNA, at a saturating NCp8 concentration (2.5 μM, 1NC per 6.8 nt) only ~ 25% annealing was measured (Figure 4D). This is in marked contrast to the NCp7 reaction, were ~ 90% of TAR2 strands were annealed at the same protein concentration.

#### tRNA^Lys3^/PBS annealing assays

**Figure 5:**
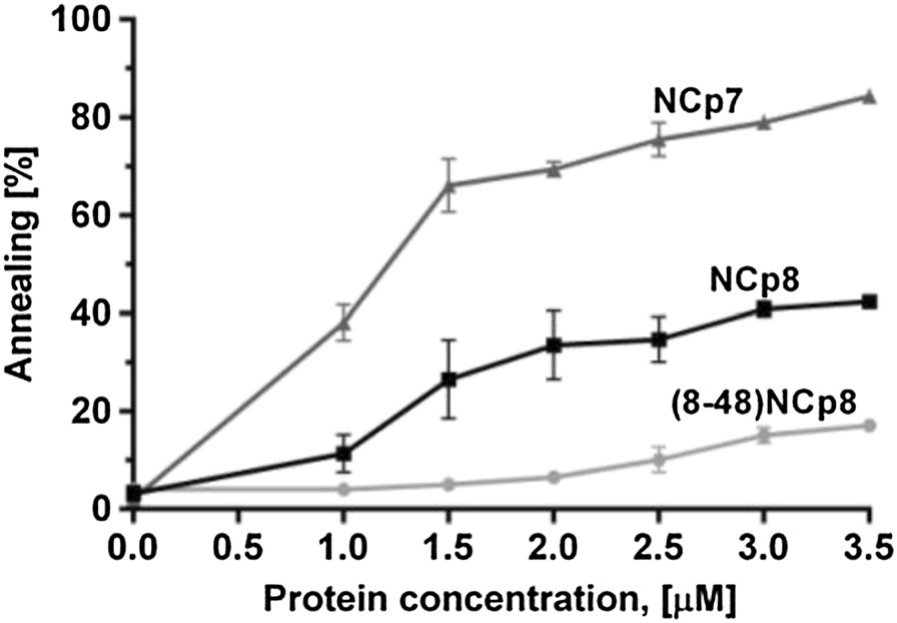
**Annealing assay using tRNA**^**Lys3 **^**and the HIV-2 PBS motif in the presence of increasing concentrations of NCp7, NCp8 or (8–48) NCp8.** The graph represents the averaged percent of annealed strands from three independent gel shift experiments for each protein. The error bars represent standard deviations.

HIV-1 and HIV-2 utilize tRNA^Lys3^ as a primer for reverse transcription and consequently both viruses posses a complementary PBS in their 5′UTRs [35]. The unmodified tRNA^Lys3^ and a 182 nt HIV-2 PBS motif transcript were used to compare NCp7 and NCp8 induced tRNA^Lys3^ annealing. We observed that NCp7 facilitated tRNA^Lys3^ annealing to the HIV-2 PBS motif significantly better than NCp8 (Figure 5). Whereas ~ 60% annealing was measured at saturating NCp7 concentration (1.5 μM, 1NC per 4.5 nt), at the same concentration of NCp8 only ~ 30% annealing was detected. Interestingly, in marked contrast to NCp7, a further increase in NCp8 concentration had only a minor effect on tRNA^Lys3^/HIV-2 PBS motif annealing, and even at a very high NCp8 concentration (3 μM) only 40% annealing was observed.

#### Role of the basic N-terminal domain in NCp8 annealing activity

To further explore the role of the NCp8 N-terminal domain, annealing assays with different DNA and RNA substrates were also performed with a truncated version of NCp8. At a saturating concentration, the (8–48) NCp8 facilitated annealing of the simplest substrate pair TAR1(−) to TAR1(+) DNA, but at reduced level (40%) comparing to NCp8 (Figure 4A). Importantly, we found that (8–48)NCp8 was completely unable to facilitate annealing of TAR1(−) DNA/TAR1 RNA, R1(−)/R1(+) DNA and TAR2 RNA/TAR2(−) DNA strands even at peptide concentration higher than saturation level (Figure 4B,C,D). The truncated NCp8 also did not promote the annealing of tRNA^Lys3^ to the HIV-2 PBS motif (Figure 5). These data, together with the sedimentation assay data presented above, strongly support the contribution of the N-terminal region in NCp8 chaperone activity.

### Strand exchange activity of NCp8 is lower than NCp7

The strand exchange assays represent a more complex approach to study the chaperone activity of a given protein. In these assays, the protein’s capacity to destabilize the structure of DNA or RNA and to enable formation of the most stable structure can be examined [22,36]. We used two types of strand exchange assays. In the first type, we tested the ability of both proteins to promote the exchange of a mismatched DNA strand in an imperfect DNA duplex by a matched DNA strand. In the second, we tested protein induced exchange of an RNA strand in an RNA/DNA heteroduplex by a complementary (+) DNA strand.

#### The DNA/DNA strand exchange assays

**Figure 6:**
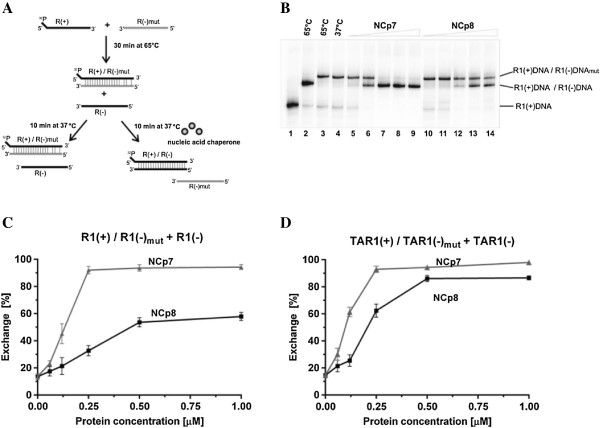
**Comparison of NCp7 and NCp8 DNA strand exchange activity. (A)** Schematic of the DNA/DNA strand exchange reaction. **(B)** A representative electrophoretic analysis of DNA strand exchange in the R1(+)DNA/R1(−) DNA_mut_ duplex at increasing concentrations of NCp7 or NCp8. Lane 1: R1(+) DNA only; lane 2: heat-annealed R1(+) DNA/R1(−)DNA; lane 3: heat-annealed R1(+) DNA/R1(−) DNA_mut_; lane 4: the heat-annealed R1(+)DNA/R1(−) DNA_mut_ duplex incubated with R1(−) DNA; lanes 5 – 9: strand exchange reaction at 0.06, 0.125, 0.25, 0.5 and 1 μM NCp7; lanes 10 – 14: strand exchange reaction at 0.06, 0.125, 0.25, 0.5 and 1 μM NCp8. **(C)** Percentage of R1(−) DNA_mut_ exchanged, measured as a ratio of perfect to imperfect duplex. **(D)** Percentage of TAR1(−) DNA_mut_ exchanged, measured as a ratio of perfect to imperfect duplex. The graphs represent the averaged data from three independent experiments. The error bars represent standard deviations.

These assays utilized three DNA oligonucleotides derived from the HIV-1 R region: R1(+), R1(−) and a mismatched R1(−)_mut_ containing seven mutations at its 3′ end. We also performed analogous assays with shorter DNA substrates corresponding to HIV-1 TAR: TAR1(+), TAR1(−) and TAR1(−)_mut_. The DNA strand exchange assays were performed similarly to those previously described [37]. Briefly, an imperfect duplex was formed by heat annealing (+) DNA and mismatched (−) DNA_mut_. Next, the duplex was incubated with fully complementary, matched (−) DNA in the presence of NCp8 or NCp7 (Figure 6A).

NCp7 effectively stimulated DNA strand exchange by replacing the mutated strand for the complementary strand in the initial, imperfect duplex in both assays, with short TAR1 (56 nt) and long R1 (96 nt) substrates (Figure 6B,C,D). We observed that NCp8 also displayed DNA strand exchange activity *in vitro*, however it was lower than NCp7. The difference between NCp7 and NCp8 was more evident in the assay with R1 substrates, where unwinding of the longer duplex and annealing of longer substrates are required for efficient strand exchange. 90% strand exchange in the R1(+)/R1(−)_mut_ DNA duplex was observed at concentrations of NCp7 even below saturating (0.25 μM, corresponding to 1 NC per 12.7 nt) (Figure 6B,C). In contrast, NCp8 in those same conditions yielded only about 30% exchange. Even at a saturating concentration of NCp8 (0.5 μM, 1 NC per 6.3) exchange level reached only ~ 50%. A further increase in NCp8 concentration of up to 1 μM (1NC per 3.2 nt) led to only minor improvement in the exchange reaction to 55% (Figure 6B,C) and 2.5 μM concentration of NCp8 was required for 80% of exchange (Additional file 1: Figure S1). In the assays with short TAR1 DNA substrates almost 90% exchange was detected at a saturating NCp7 concentration (0.25 μM, 1NC per 7.4 nt), but at the same concentration of NCp8, only ~ 60% exchange was measured (Figure 6D). However, in this case, a further increase of NCp8 concentration led to a significant activation of the exchange reaction.

#### The RNA/DNA strand exchange assays

**Figure 7:**
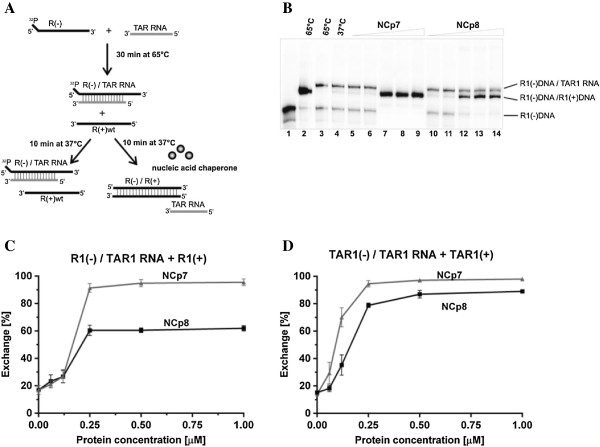
**Comparison of NCp7 and NCp8 RNA strand exchange activity. (A)** A schematic of the RNA/DNA strand exchange reaction. Note that the TAR1 RNA may adopt a stem-loop structure, not shown here. **(B)** A representative electrophoretic analysis of RNA strand exchange in the R1(−) DNA/TAR1 RNA duplex at increasing concentrations of NCp7 or NCp8. Lane 1: R1(−) DNA only; lane 2: heat-annealed R1(−) DNA/TAR1 RNA; lane 3: heat-annealed R1(−)DNA/R1(+)DNA; lane 4: heat-annealed R1(−) DNA/TAR1 RNA incubated with R1(+)DNA at 37°C; lanes 5 – 9: strand exchange reaction at 0.06, 0.125, 0.25, 0.5 and 1 μM NCp7; lanes 10 – 14: strand exchange reaction at 0.06, 0.125, 0.25, 0.5 and 1 μM NCp8. **(C)** Percentage of TAR1 RNA exchanged, measured as a ratio of R1(−) DNA/R1(+)DNA to R1(−) DNA/TAR1 RNA duplex. **(D)** Percentage of TAR1 RNA exchanged, measured as a ratio of TAR1(−) DNA/TAR1(+) DNA to TAR1(−) DNA/TAR1 RNA duplex. The graphs represent averaged data from three independent experiments. The error bars represent standard deviations.

Further assays aimed to determine the ability of HIV-2 nucleocapsid protein to stimulate RNA strand exchange in a preformed RNA/DNA duplex (Figure 7A). These experiments were designed and performed similarly to the DNA exchange assays with R1 and TAR1 substrates described above, but TAR1 RNA (56 nt) was used instead of mismatched (−) DNA strands. We found that NCp8 promoted RNA strand exchange less effectively than NCp7 and the difference in the proteins’ activity was more evident in the assays with the longer R1 substrates than with TAR1 oligonucleotides. In these two assays, the initial RNA/DNA heteroduplexes are the same length (the polyA sequence is not involved in duplex formation), but the length of final duplexes differ: TAR1(−)/TAR1(−) is 56 bp, while R1(−)/R1(+) is 96 bp. The exchange level of TAR1 RNA for R1(+) in the initial R1(−) DNA/TAR1 RNA duplex was ~ 95% at 0.25 μM NCp7 (1NC per 11 nt), whereas only ~ 60% was observed for NCp8 (Figure 7B, C). NCp8, even at levels above saturation (1 μM; 1NC per 2.75 nt), did not stimulate RNA strand exchange in the R1(−) DNA/TAR1 RNA. However, in the assay with shorter oligonucleotides (Figure 7D), NCp8 significantly increased exchange of TAR1 RNA for TAR1(+) DNA in the TAR1(−) DNA/TAR1 RNA heteroduplex at a saturating concentration (0.125 μM). As a result of protein mediated strand exchange, a thermodynamically less stable DNA/DNA duplex was formed. An RNA/DNA heteroduplex is more stable than the analogous DNA/DNA duplex. The calculated Tm for TAR1(−) DNA/TAR1 RNA is 84.8°C, whereas it is 75.5°C for the TAR1(−) DNA/TAR(+) DNA duplex [38]. However, a minimum free energy state of the entire system differs.

## Discussion and conclusions

In this work, the nucleic acid chaperone activity of two nucleocapsid proteins, HIV-1 (NCp7) and HIV-2 (NCp8), were compared *in vitro*. The activity of NCp7 has been extensively studied, however diversity in the approaches and conditions used prompted us to simultaneously assay both nucleocapsid proteins. We found it important to directly compare the aggregation, annealing and strand exchange activities of these two related NC proteins.

Sequence non-specific aggregation of NA leading to an increase in local concentration is considered a major mechanism of NC-induced NA annealing [11,23,30]. An electrostatic model of NCp7-induced NA aggregation has been proposed and the highly cationic N-terminal domain of NCp7 is thought to be crucial for both NA aggregation and annealing activity [11,12,30]. The truncated NCp7 peptide (12–55) NCp7 or the NCp7 cationic N-terminal pentamutant (with all 5 basic residues in the N-terminal region replaced with alanines) did not cause nucleic acid aggregation or chaperone the annealing of HIV-1 TAR(−) DNA/TAR RNA at saturating protein concentrations [12,31]. Moreover, mutation of three of five basic residues in the N-terminus strongly impaired NCp7 activity [31]. Despite the fact that the N-terminal domain of NCp8 is shorter and contains only three positively charged amino acid residues, both NCp7 and NCp8 display similar NA aggregation activities and TAR1(−) DNA/TAR1 RNA annealing levels (Figures 3 and 4B). Basic residues distributed in other NCp8 regions, such as the linker and ZFs, may also participate in protein activity. Since the N-terminal segment of NCp8 is shorter and the flexibility of the basic linker region is limited, it has been proposed that the hydrophobic cleft in the second ZF of NCp8 plays a role similar to that of the N-terminal region of NCp7 in nonspecific NA recognition [16,17]. Although we cannot exclude these regions, our results obtained with the (8–48) NCp8 peptide, which displays reduced aggregation activity (Figure 3) and was unable to facilitate annealing of most NA substrates (Figures 4B-D and 5), strongly support the crucial role of basic N-terminal segment in NCp8 chaperone activity.

The chaperone activity of retroviral nucleocapsid proteins plays an important role in facilitating remodelling of nucleic acid strands during reverse transcription [39]. We performed several gel shift assays to examine the ability of both proteins to chaperone nucleic acids annealing and strand exchange in duplex nucleic acid structures. Annealing of HIV-1 TAR(−) DNA to a complementary TAR RNA or TAR(+) DNA is often use as model assay to study the NA chaperone activity of proteins. Spontaneous formation of the TAR1(−) DNA/TAR1 RNA duplex *in vitro* is extremely slow at physiological conditions, however addition of NCp7 accelerates the annealing rate about 3000-fold [22,32]. The mechanism of HIV-1 TAR hairpins annealing has been extensively studied and it was determined that NCp7 switches the inefficient loop – loop pathway to the zipper pathway, leading to an efficient annealing reaction [40-42]. Although TAR is considered of major importance in first strand transfer, the transferred (−)ssDNA consists of the entire R (TAR, polyA) and U5 sequence. Moreover, in the 3′ R region, in addition to TAR, a shortened polyA hairpin is present and stabilization of the polyA hairpin in the 5′ and 3′ R regions inhibited strand transfer in HIV-1 [20]. We found that NCp8 and NCp7 facilitate TAR1(−) DNA/TAR1(+) DNA and TAR1(−) DNA/TAR1 RNA annealing with high, comparable efficiencies (Figure 4A,B). However, NCp8 is less efficient in the annealing of longer substrates corresponding to the entire HIV-1 R region (Figure 4C). These data suggest that the additional sequence, which is able to form a shortened polyA hairpin, inhibits NCp8-induced annealing.

In view of this observation, it was interesting to test the ability of NCp8 to facilitate annealing of its cognate TAR2 hairpins. Present at the 5′ end of the HIV-2 genome, TAR2 (Figure 2) is more complex and may exist in two highly structured forms: a three-hairpin branched form [34] and an extended form with two hairpins [33]. Structural *in silico* analysis of the HIV-2 RNA 3′end R region [43], supported by a recently published structure of the closely related SIV_MAC_ genome [44], indicates that the 3′ TAR2 RNA may adopt the stable three-hairpin form. In contrast to NCp7, NCp8 facilitates the *in vitro* annealing of TAR2 RNA/TAR2(−) DNA only very weakly at saturating concentrations (Figure 4D). Numerous studies have been dedicated to explaining the relationship between nucleic acid structure and NC mediated first strand transfer. The structure and the degree of thermostability of nucleic acid substrates are proposed to be major factors influencing NC-chaperoned first strand transfer [36,39,45]. NCp7 displays only a weak destabilization activity, which strongly depends on the stability of the NA. Additionally, the local structure of the (−)ssDNA (bulges, internal loops) and the stability of the acceptor RNA are both critical [20,24,46,47]. Results from annealing assays performed with diverse RNA and DNA oligonucleotides suggest that NCp8 chaperone activity is limited by the stability and length of the substrates to a greater degree than that of NCp7.

Important confirmation of this observation was obtained in the strand exchange assays (Figures 6 and 7). Strand exchange activity is the result of the combined action of both helix destabilization and annealing activities [9,22,48]. The DNA and RNA strand exchange assays with shorter substrates demonstrate that NCp8 displays DNA and RNA strand exchange activity, but that a higher concentration relative to that of NCp7 is required for effective exchange (Figure 7C,D). With longer or more stable substrates, a significant decrease in exchange efficiency was observed in the presence of NCp8, but not NCp7. The weaker activity of NCp8 in the DNA and RNA exchange assays with the R1 substrates, compared to that with the shorter TAR1 substrates, may be the result of lower helix destabilization and/or lower annealing activity. More advanced biophysical studies are needed to determine the details of NCp8 mediated destabilization.

It is generally accepted that the mechanism of strand exchange depends on chaperone protein induced destabilization of a nucleic acid duplex and formation of a more thermodynamically stable duplex with another nucleic acid strand where complementarities are more extended [9,22,48]. Although this statement applies to our DNA strand exchange assays, the ability of both NCp7 and NCp8 to promote the exchange of TAR1 RNA for TAR1(+) DNA in the initial heteroduplex may seem surprising, as the final duplex is a thermodynamically less stable form. However, chaperone proteins always drive the entire system to a minimum free energy state [49] and the TAR1 RNA released during this strand exchange may form a very stable hairpin (ΔG = − 25.7 kcal/mol). Free energy calculation shows that the overall free energy of the final products is indeed lower (TAR1 RNA ΔG = −25.7 kcal/mol and TAR1(−) DNA/TAR1(+) DNA ΔG = − 62.06 kcal/mol) than that of the reactant system (TAR1(+) DNA ΔG = −10.04 kcal/mol and TAR(−) DNA/TAR1 RNA ΔG = − 69.6 kcal/mol). Thus, the exchange effect observed arises from the capacity of NC to lower the overall system free energy and thereby release the TAR1 RNA from the DNA/RNA hybrid.

A prominent annealing reaction in the HIV replication cycle is the placement of tRNA^Lys3^ onto the primer binding site sequence (PBS) located in the 5′ UTR of the viral RNA. It is not definitively established whether it is the NC domain of Gag or the mature NC protein that is crucial for this process in infected cells or virions. Recent studies show that the initial tRNA^Lys3^ annealing is probably promoted by Gag, whereas the final tRNA^Lys3^/vRNA remodeling step is facilitated by the mature NC [5,50,51]. Numerous studies have demonstrated that *in vitro*, NCp7 chaperones tRNA^Lys3^ annealing onto the HIV-1 PBS motif very efficiently. Interestingly, we found that NCp8 very inefficiently facilitated *in vitro* annealing of tRNA^Lys3^ to its cognate PBS motif (Figure 5). Additional interactions between tRNA^Lys3^ and HIV-1 RNA outside the PBS have been identified as crucial for viral replication [52] and other factors, such as cellular RNA helicase A [53], lysyl-tRNA synthetase [54] or viral Vif protein [55] may play a role in tRNA^Lys3^ placement. The tRNA^Lys3^/HIV-2 RNA complex has not been investigated as extensively as that of HIV-1, however structural studies of the heat-annealed complex supported by mutational analysis showed interactions occurring in other secondary structure elements outside of the acceptor stem of tRNA^Lys3^ and the PBS [56,57]. Since the HIV-2 PBS motif has a more complex structure and NCp8 appears to be weaker chaperone, additional factors may be even more important for tRNA^Lys3^/HIV-2 PBS annealing than for HIV-1.

In conclusion, our *in vitro* data suggest that the HIV-2 NC protein displays reduced nucleic acid chaperone activity compared to that of HIV-1 NC. We found that NCp8 activity is limited by substrate length and stability to a greater degree than that of NCp7. This is especially interesting in light of the fact that the HIV-2 5′UTR structure is more complicated than that of HIV-1 [33,34,58]. Our assays with substrates derived from the HIV-2 genome showed important differences between NCp8 and NCp7. Our data indicate that to better understand the biological relevance of chaperone proteins from different retroviruses, studies using cognate RNA and DNA fragments should be considered. The lower chaperone activity observed with NCp8 may influence the efficiency of reverse transcription and other key steps of the HIV-2 replication cycle. Additionally, other viral or cellular proteins may be engaged in chaperoning these processes in HIV-2. As crucial factors in retroviral replication, NC proteins are often proposed as being promising targets for antiretroviral therapy [59,60], therefore it is important that their properties are studied extensively. We believe that the above results will contribute to understanding the differences between retroviral nucleocapsid proteins and in the long term, between HIV-2 and the more pathogenic HIV-1.

## Methods

### Proteins

NCp7 and NCp8 proteins were purified as recombinant glutathione *S-*transferase (GST) fusion proteins from *E. coli* BL21—Codon Plus (DE3)—RIL cells (Stratagene), based on engineered expression vectors pGEX-4 T-3-NCp7 and pGEX-4 T-3-NCp8 [58]. The HIV-1_NL4–3_ and HIV-2_ROD_ nucleocapsid protein coding sequences*coli* BL21—Codon Plus (DE3)—RIL cells (Stratagene), based on engineered expression vectors pGEX-4 T-3-NCp7 and pGEX-4 T-3-NCp8 [58]. The nucleocapsid protein coding sequences of HIV-1_NL4–3_ and HIV-2_ROD_ were taken from http://ncbi.nlm.nih.gov. GST-tagged proteins were purified by affinity chromatography on Glutathione-Sepharose (Amersham Pharmacia Biotech.) and the GST tag was cleaved off with thrombin enzyme. The proteins were further purified on a Superdex 200 FPLC column, and molecular masses were determined using MALDI TOF (Autoflex, Bruker Daltonics). The purified proteins were lyophilized and stored at −80°C. Synthetic (8–48) NCp8 peptide was obtained from ThermoFischer Scientific. Proteins were dissolved in freshly prepared, oxygen-free buffer containing 30 mM HEPES pH 6.5, 30 mM NaCl and 0.1 mM ZnCl_2_. Activity of each protein preparation was compared with the activity of the synthetic full-length (48aa) NCp8 (Additional file 2: Figure S2).

### DNA and RNA substrates

ODNs corresponding to the TAR and the R sequences of HIV-1_MAL_ or HIV-2_ROD_ were purchased from Genomed (Poland). Oligonucleotide sequences are presented in Table 1. TAR1(−)_mut_ DNA and R1(−)_mut_ DNA correspond to HIV-1 antisense TAR or R, but each contain 7 mismatches at the 3′ end (Table 1, residues shown in italic). R1(−)_mut_ DNA was designed based on [37]. RNA oligonucleotides were synthesized with the Ambion T7-MEGAshortscript following the manufacturer’s protocol, based on PCR generated templates. Transcripts were purified by denaturing gel electrophoresis (8 M urea) in 1× TBE, followed by elution and ethanol precipitation. Purified RNA was dissolved in sterile water and stored at −20°C. TAR and R ODNs were ^32^P-labelled at the 5′-end with [γ-^32^P]ATP using T4 polynucleotide kinase (Fermentas) according to manufacturer’s protocol and were purified using NucAway Spin Columns (Life Technologies). The *in vitro* synthesized, unmodified human tRNA^Lys3^ (hereafter referred as tRNA^Lys3^) was 3′-end labelled using [α-^32^P] pCp and T4 RNA ligase, based on standard protocol.

**Table 1:** The sequences of TAR and R oligonucleotides used in this study

**Name**	**Sequence 5′ ‒ 3′‒**	**Length**
TAR1(+) DNA	GGTCTCTCTTGTTAGACCAGGTCGAGCCCGGGAGCTCTCTGGCTAGCAAGGAACCC	56
TAR1(−) DNA	GGGTTCCTTGCTAGCCAGAGAGCTCCCGGGCTCGACCTGGTCTAACAAGAGAGACC	56
TAR1(−)_mut_ DNA	GGGTTCCTTGCTAGCCAGAGAGCTCCCGGGCTCGACCTGGTCT*TTG*AA*CTC*AGA*GG*	56
TAR1 RNA	GGUCUCUCUUGUUAGACCAGGUCGAGCCCGGGAGCUCUCUGGCUAGCAAGGAACCC	56
R1(+) DNA	GGTCTCTCTTGTTAGACCAGGTCGAGCCCGGGAGCTCTCTGGCTAGCAAGGAACCCACTGCTTAAGCCTCAATAAAGCTTGCCTTGAGTGCCTCCC	96
R1(−) DNA	GGGAGGCACTCAAGGCAAGCTTTATTGAGGCTTAAGCAGTGGGTTCCTTGCTAGCCAGAGAGCTCCCGGGCTCGACCTGGTCTAACAAGAGAGACC	96
R1(−)_mut_ DNA	GGGAGGCACTCAAGGCAAGCTTTATTGAGGCTTAAGCAGTGGGTTCCTTGCTAGCCAGAGAGCTCCCGGGCTCGACCTGGTCTAACA*TC*AG*TCT*C*TA*	97
TAR2 RNA	GGTCGCTCTGCGGAGAGGCTGGCAGATTGAGCCCTGGGAGGTTCTCTCCAGCACTAGCAGGTAGAGCCTGGGTGTTCCCTGCTAGACTCTCACCAGCACTTGGCCGGTGCTGGGCAGACGGCC	123
TAR2(−) DNA	GCCGTCTGCCCAGCACCGGCCAAGTGCTGGTGAGAGTCTAGCAGGGAACACCCAGGCTCTACCTGCTAGTGCTGGAGAGAACCTCCCAGGGCTCAATCTGCCAGCCTCTCCGCAGAGCGAC	121

### TAR and R oligonucleotides annealing assays

Refolded ^32^P-labelled antisense DNA oligonucleotide (1 nM) and complementary, unlabelled sense DNA or RNA (6 nM) were incubated with increasing protein concentrations (0 – 0.5 μM) in buffer A (20 mM Tris–HCl, pH 7.2, 30 mM NaCl, 0.1 mM MgCl_2,_ 10 μM ZnCl_2_ and 5 mM DTT) at 37°C for 5 minutes. Subsequently, the reactions (10 μl) were chilled on ice and quenched with 5 μl of Stop Solution (20% glycerol, 20 mM EDTA pH 8.0, 0.2% SDS, 0.25% bromophenol blue and 0.4 mg/ml yeast tRNA), to denature proteins and induce their release from the oligonucleotides. Samples were analyzed by native PAGE (8%) in 0.5× TBE at 4°C (DNApointer, Biovectis).

### tRNA^Lys3^/PBS annealing assays

Prior to the annealing reaction, the ^32^P-labelled tRNA^Lys3^ was refolded in 50 mM Hepes (pH 7.5) by heating at 85°C for 2 minutes and slow cooling to 60°C, followed by addition of MgCl_2_ to 10 mM and placement on ice. The +197 – 379 HIV-2 transcript (hereafter referred to as HIV-2 PBS motif) was refolded in 50 mM Hepes, pH 7.5 by heating at 85°C for 2 minutes and slow cooling to 60°C, followed by addition of MgCl_2_ to 10 mM. Subsequently RNA was incubated at 37°C for 10 minutes and placed on ice. Refolded tRNA^Lys3^ was combined with refolded +197 – 379 HIV-2 RNA in a solution containing 50 mM Hepes, pH 7.5, 20 mM NaCl, 5 mM DTT, and 1 mM MgCl_2_. Initially, 10 nM tRNA^Lys3^ was combined with 25 nM HIV-2 PBS motif and incubated at 37°C for 10 minutes. Upon addition of protein, the annealing reaction proceeded at 24°C for 30 minutes. Aliquots from the annealing reaction were quenched by incubation with 1% (w/v) SDS at room temperature for 10 minutes. The samples were phenol/chloroform-extracted twice, mixed with loading dye and separated on an SDS-10% polyacrylamide gel in 1 × TBE at room temperature.

### DNA/DNA strand exchange assays

^32^P-labelled R1(+), R1(−) and R1(−)_mut_ ODNs were separately heat denatured for 5 minutes at 95°C in water and chilled on ice. All components were kept at 4°C. Subsequently, 1 nM ^32^P-labelled R1(+) and 5 nM R1(−)_mut_ were mixed with buffer A to a final concentration of 20 mM Tris–HCl, pH 7.2, 30 mM NaCl, 0.1 mM MgCl_2_, 10 μM ZnCl_2_ and 5 mM DTT. Reactions were incubated for 30 minutes at 65°C to form the R1(+)/R1(−)_mut_ duplex and chilled on ice. Subsequently 5 nM R1(−) and varying concentrations of protein (0–1 μM) were added. Samples were incubated for 10 minutes at 37°C, chilled on ice and quenched with 5 μl of Stop Solution. Samples were analyzed by 8% native PAGE in 0.5× TBE at 4°C. A DNA/DNA strand exchange assay with ^32^P-labelled TAR1(+), TAR1(−) and TAR1(−)_mut_ was performed similar to that described above.

### RNA/DNA strand exchange assays

^32^P-labelled R1(−), R1(+) and TAR1 RNA oligonucleotides were separately heat denatured for 5 minutes at 95°C in water and chilled on ice. All components were kept at 4°C. Subsequently, 1 nM ^32^P-labelled R1(−) and 5 nM TAR1 RNA, were mixed with reaction buffer A to a final concentration of 20 mM Tris–HCl, pH 7.2, 30 mM NaCl, 0.1 mM MgCl_2_, 10 μM ZnCl_2_ and 5 mM DTT. Reactions were incubated for 30 minutes at 65°C to form the R1(−)/TAR1 RNA duplex and chilled on ice. Subsequently, 5 nM R1(+) and varying concentrations of protein (0–1 μM) were added. Samples were incubated for 10 minutes at 37°C, chilled on ice and quenched with 5 μl of Stop Solution. An RNA/DNA strand exchange assay with ^32^P-labelled TAR1(−), TAR1 RNA and TAR1(+) was performed similar to that described above. Calculations of the hybridization thermodynamics were made using HyTher server [38].

### Sedimentation assays

10 nM ^32^P-labelled TAR1(−) was combined with 40 nM complementary unlabelled TAR1(+) in a buffer containing 50 mM HEPES pH 7.5, 20 mM NaCl, and 0.2 mM MgCl_2_. Diverse amounts of NCp8 were added (0–2 μM) and reactions (10 μl) were incubated at 37°C for 30 minutes. Subsequently, the mixtures were centrifuged at 11400 rpm for 20 minutes. Supernatants (2 μl) were collected and subjected to scintillation counting.

All gels were autoradiographed and quantitatively analyzed by phosphorimaging using FLA-5100 phosphorimager with MultiGaugeV 3.0 software (FujiFilm).

In all cases, at least three independent experiments were performed, and the data presented are representative of the whole.

## Abbreviations

DTT: Dithiothreitol; HIV-1: Human immunodeficiency virus type 1; HIV-2: Human immunodeficiency virus type 2; NA: Nucleic acid; NAC: Nucleic acid chaperone; NC: Nucleocapsid protein; NCp7: HIV-1 nucleocapsid protein; NCp8: HIV-2 nucleocapsid protein; TAR: *Trans*-activation response element; ZF: Zinc finger.

## Competing interests

The authors declare that they have no competing interest.

## Authors’ contributions

KPW and AS performed the experiments. KPW and KJP conceived and designed the experiments, analyzed and interpreted the data, and wrote the manuscript. All authors read and approved the final manuscript.

## Supplementary Material

Additional file 1 **The NCp8 DNA strand exchange activity.** (A) A representative electrophoretic analysis of DNA strand exchange in the R1(+) DNA/R1(-) DNAmut duplex at increasing concentrations of NCp8. Lane 1: R(+) DNA only; lane 2: heat-annealed R1(+) DNA/R1(-) DNA; lane 3: heat-annealed R1(+) DNA/R1(-) DNAmut; lane 4 – 9: strand exchange reaction at 0.5, 1, 1.5, 2, 2.5, 3 μM NCp8. (B) Percentage of R1(-) mut exchanged, measured as a ratio of perfect to imperfect duplex. Assays were performed as described in Methods section. The graph represents the averaged data from three independent experiments. The error bars represent standard deviations. Click here for file

Additional file 2 **Comparison of the annealing (left) and strand exchange (right) activities of recombinant NCp8 proteins from three independent preparations (NCp8 1, NCp8 2, NCp8 3) and chemically synthesized NCp8 (NCp8chem).** The annealing assays were performed with TAR1(-) DNA and TAR1(+) DNA substrates. The DNA strand exchange activity was tested in the assays with R1(+) DNA, R1(-) DNAmut and R1(-) DNA substrates. Assays were performed as described in Methods section. The graphs represent the averaged data from three independent experiments. The error bars represent standard deviations. Click here for file
