# Supplementary material for: Similarities and differences in the nucleic acid chaperone activity of HIV-2 and HIV-1 nucleocapsid proteins in vitro
Source: Retrovirology. 2014 Jul 3;11:54. doi: 10.1186/1742-4690-11-54 (PMC4227088; doi:10.1186/1742-4690-11-54)
Supplement: Additional file 1 — The NCp8 DNA strand exchange activity. (A) A representative electrophoretic analysis of DNA strand exchange in the R1(+) DNA/R1(-) DNAmut duplex at increasing concentrations of NCp8. Lane 1: R(+) DNA only; lane 2: heat-annealed R1(+) DNA/R1(-) DNA; lane 3: heat-annealed R1(+) DNA/R1(-) DNAmut; lane 4 – 9: strand exchange reaction at 0.5, 1, 1.5, 2, 2.5, 3 μM NCp8. (B) Percentage of R1(-) mut exchanged, measured as a ratio of perfect to imperfect duplex. Assays were performed as described in Methods section. The graph represents the averaged data from three independent experiments. The error bars represent standard deviations. [file 1742-4690-11-54-S1.docx]

**Figure S1.**

| **A B**  **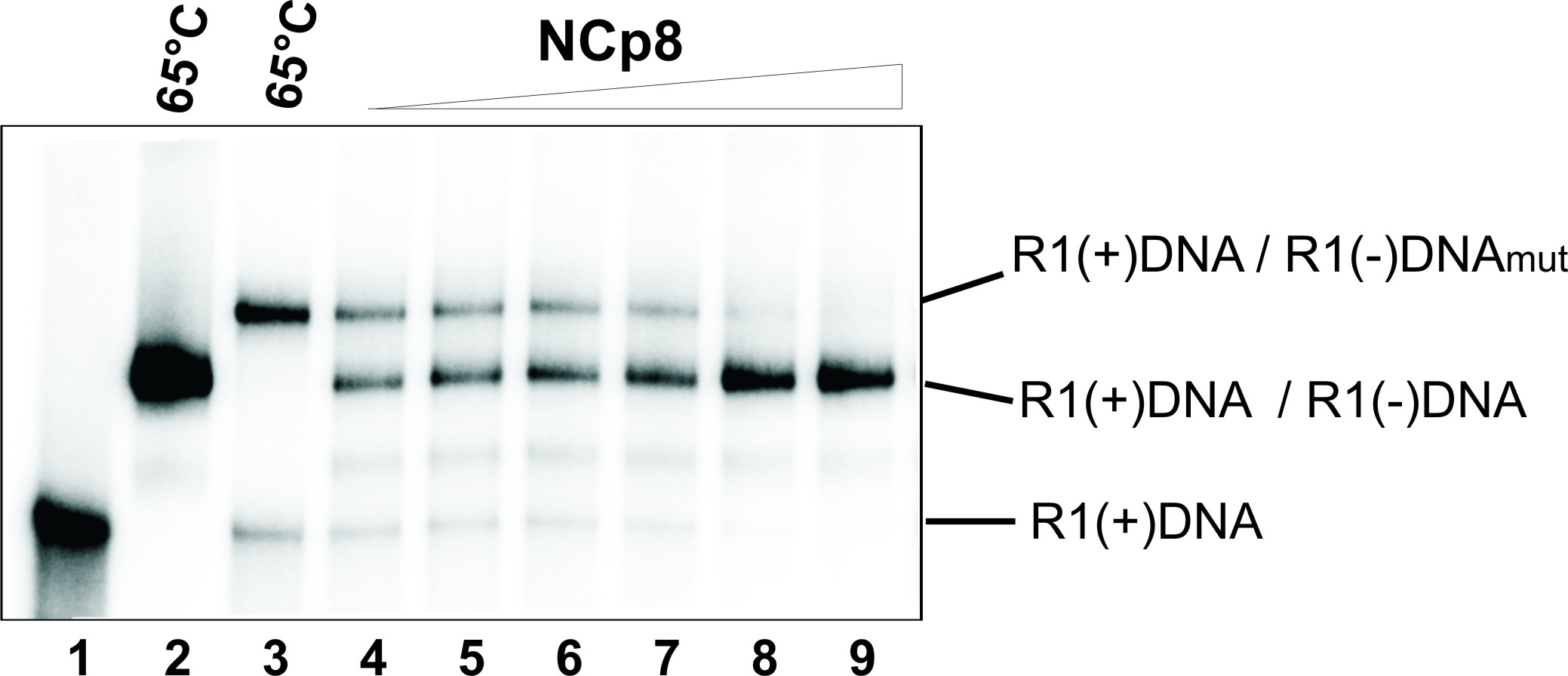** | **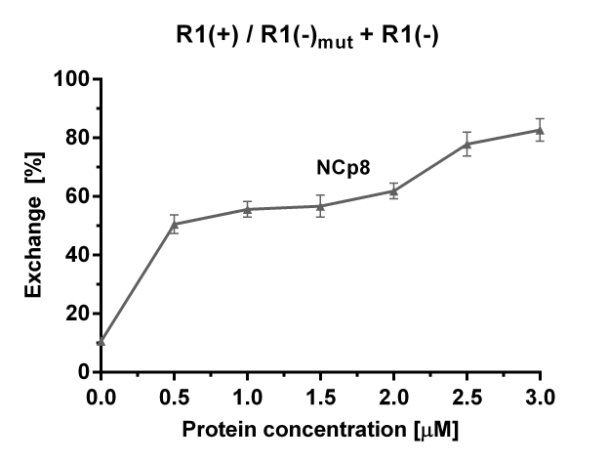**  **R1(+)DNA / R1(-)DNA_mut_ +R1(-)DNA** |
| --- | --- |

The NCp8 DNA strand exchange activity. (A) A representative electrophoretic analysis of DNA strand exchange in the R1(+)DNA / R1(-)DNA_mut_ duplex at increasing concentrations of NCp8. Lane 1: R(+)DNA only; lane 2: heat-annealed R1(+)DNA / R1(-)DNA; lane 3: heat-annealed R1(+)DNA / R1(-)DNA_mut_; lane 4 – 9: strand exchange reaction at 0.5, 1, 1.5, 2, 2.5, 3 µM NCp8. (B) Percentage of R1(-)_mut_ exchanged, measured as a ratio of perfect to imperfect duplex. Assays were performed as described in Materials and Methods section. The graph represents the averaged data from three independent experiments. The error bars represent standard deviations.
