# Supplementary material for: Similarities and differences in the nucleic acid chaperone activity of HIV-2 and HIV-1 nucleocapsid proteins in vitro
Source: Retrovirology. 2014 Jul 3;11:54. doi: 10.1186/1742-4690-11-54 (PMC4227088; doi:10.1186/1742-4690-11-54)
Supplement: Additional file 2 — Comparison of the annealing (left) and strand exchange (right) activities of recombinant NCp8 proteins from three independent preparations (NCp8 1, NCp8 2, NCp8 3) and chemically synthesized NCp8 (NCp8chem). The annealing assays were performed with TAR1(-) DNA and TAR1(+) DNA substrates. The DNA strand exchange activity was tested in the assays with R1(+) DNA, R1(-) DNAmut and R1(-) DNA substrates. Assays were performed as described in Methods section. The graphs represent the averaged data from three independent experiments. The error bars represent standard deviations. [file 1742-4690-11-54-S2.docx]

**Figure S2.**

| **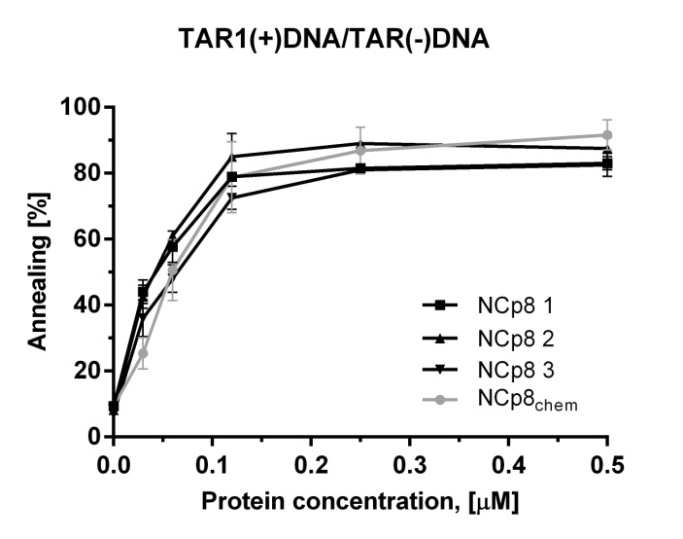**  **TAR1(+)DNA / TAR1(-)DNA R1(+)DNA / R1(-)DNA_mut_ + R1(-)DNA** | **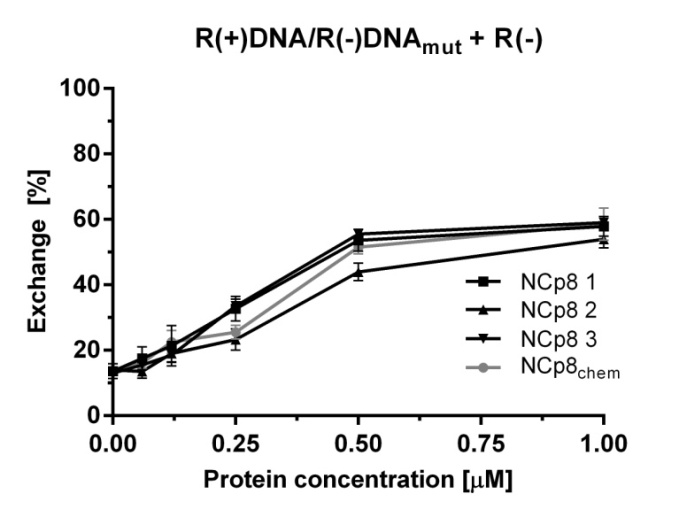** |
| --- | --- |

Comparison of the annealing (left) and strand exchange (right) activities of recombinant NCp8 proteins from three independent preparations (NCp8 1, NCp8 2, NCp8 3) and chemically synthesized NCp8 (NCp8_chem_). The annealing assays were performed with TAR1(-)DNA and TAR1(+) DNA substrates. The DNA strand exchange activity was tested in the assays with R1(+)DNA, R1(-)DNA_mut_ and R1(-)DNA substrates. Assays were performed as described in Materials and Methods section. The graphs represent the averaged data from three independent experiments. The error bars represent standard deviations.
